# Serum Levels of Anti-Hepatitis B Surface Antibody Among Vaccinated Population Aged 1 to 18 Years in Ahvaz City Southwest of Iran

**DOI:** 10.5812/hepatmon.13625

**Published:** 2014-01-12

**Authors:** Reza Norouzirad, Abdol Hussein Shakurnia, Mohammad-Ali Assarehzadegan, Amirarsalan Serajian, Mehdi Khabazkhoob

**Affiliations:** 1 Biochemistry Department, Faculty of Medicine, Dezful University of Medical Sciences, Dezful, IR Iran; 2 Health Research Institue, Infectious and Tropical Diseases Research Center, Immunology Department, Faculty of Medicine, Ahvaz Jundishapur University of Medical Sciences, Ahvaz, IR Iran; 3 Immunology Department, Faculty of Medicine, Ahvaz Jundishapur University of Medical Sciences, Ahvaz, IR Iran; 4 Health Education Research Group, Jahade Daneshgahi of Khouzestan, Ahvaz, IR Iran; 5 Epidemiology Department, Faculty of Health, Shahid Beheshti University of Medical Sciences, Tehran, IR Iran

**Keywords:** Hepatitis B, Vaccination, Iran

## Abstract

**Background:**

The duration of protection following primary series vaccination against hepatitis B is unknown in children and adolescents. It has been shown that the level of anti-hepatitis B surface antigen antibodies (anti HBs Ab) declines over years after vaccination.

**Objectives:**

The aim of this study was to estimate the long-term immunity against hepatitis B virus infection among children and adolescents who had received a complete hepatitis B vaccination series during infancy.

**Patients and Methods:**

In a cross-sectional study, the - anti-HBsAb levels of 840 vaccinated children and adolescents were determined by enzyme-linked immunosorbent assay.

**Results:**

Hepatitis B seroprotection rates (anti HBsAb ≥ 10 IU/L) among vaccinated children and adolescents aged 1 and 18 years were 90% and 48.9%, respectively. The declining trend of geometric mean titer of anti-HBsAb levels was observed as changed from 272.3 IU/L to 94.1 IU/L in 1 and 18-year-old population, respectively. A significant negative correlation was found between age and anti-HBsAb levels (r = - 0.220, P = 0.0001).

**Conclusions:**

The results showed a declining trend in anti-HBsAb titers over the time after vaccination against hepatitis B virus in our region. Further studies are warranted to establish the need for a booster dose in cases that are at risk of hepatitis B virus infection.

## 1. Background

Hepatitis B virus (HBV) infection is a major worldwide health problem, especially in Asia. There are about 350 million chronic carriers of HBV in the world. HBV is known to be the major cause of liver failure, cirrhosis, and hepatocellular carcinoma (1-3).

It has been estimated that more than one third of the population in the world has been infected with HBV. The epidemiological studies have shown that about 35% of Iranians have been exposed to HBV and 3% are chronic carriers, ranging from 1.7% to 5.1% in Fars and Golestan provinces, respectively (4-6). Therefore, HBV is an important candidate for public health measures for prevention, early diagnosis, and treatment (7).

Universal immunization against HBV is considered to be the best way of prevention of HBV infection. Due to the importance of HBV infection in Iran, the National HBV Vaccination Program has been included in the Expanded Programme on Immunization (EPI) which was started at 1993 by a recombinant vaccine. The adopted schedule by the Iranian Ministry of Health was three doses of a recombinant HBV vaccine (Heberbiovac Cuba: Heber Biotec S.A., Havana, Cuba) administered to all infants at the ages of 0, 2 and 6 months to coincide with other compulsory vaccines. One study from Iran in reported in 2011 that coverage rate of HBV vaccination in children was more than 95.0%, where the infants had received 3 doses of recombinant vaccines (8).

The main criterion for immunity was appropriate concentration of anti-HBsAb in serum. Greater levels of antibody production would lead to better immunity. Serum hepatitis B surface antigen (HBsAg) was considered as a marker of chronic HBV infection; and anti-HBsAb levels of ≥ 10 IU/L indicated the protective immunity (9, 10).

Duration of protection against HBV after hepatitis B vaccination depends on presence of anti-HBsAb levels in serum. The results of various studies revealed that higher concentrations of serum antibody might lead to longer duration of immunity, but the duration was unknown (11-13).

The rapid decline in anti-HBsAb levels in children and adolescents, which makes the issue about the survival of vaccine-induced immunity in this age group would be discussed (14, 15).

Increased sexual activity and risky behavior will increase the risk of HBV infection. Therefore, vaccine-induced immunity should be continued until puberty and thereafter (16, 17). Thus, a booster dose of the vaccine may be necessary if HBV immunity wipes out during this period.

## 2. Objectives

As there was scarce data about the long-term persistence of anti-HBs Abs after vaccination in our region, this study was designed to determine the levels of anti-HBsAb and immunity to HBV among vaccinated children and adolescents in Ahvaz, a city located in southwestern Iran.

## 3. Patients and Methods

In a cross-sectional study, 840 healthy individuals (1-18 years old) were selected by multistage cluster sampling from health care units and medical centers in Ahvaz between March and September 2011.

Considering an expected prevalence of anti-HBsAb of 90% in target groups, the sample size was estimated. Based on this prevalence in the study groups, with α = 0.02 and desired precision equal to 0.05, statistical analysis indicated that 864 sera were required. The sera were collected from healthy subjects whom were referred to health care units and medical centers in Ahvaz.

Blood samples were taken after signing an informed consent. Then the serum samples were collected and stored at -20˚C. All participants were basically healthy, with no acute or chronic illnesses. Subjects with a history of recent infectious contagious diseases, any immune compromising conditions and dialysis or thalassemia were excluded. We also excluded any subject in the study that was found to have positive test results for HBsAg and/or anti-hepatitis B core antigen antibodies (anti-HBcAb). Finally, 840 samples were tested.

At the time of specimen collection, information regarding date of birth, sex, health status, and dates of vaccinations was recorded. Sera from the subjects were tested by commercial microplate enzyme-linked immunosorbent assay kits (HBsAb, HBsAg and HBcAb, DIA.PRO, Italy) according to the manufacturer’s instructions. We considered cases as seropositive against HBV, if anti-HBsAb levels were equal or greater than 10 IU/L, and seronegative if anti-HBsAb levels were less than 10 IU/L.

All samples were categorized to three groups according to anti-HBsAb levels: 1) Non responders or seronegatives (< 10 IU/L), 2) Low responders (10-100 IU/L) and 3) Good responders (> 100 IU/L) (12).

All participants were divided into 4 groups by their age according to a previous study as less than six years; from 6 to 10 years; from 11 to 15 years; and more than 16 years (18).

All data were summarized as mean and standard deviation for continuous variables and as, frequency and percentage for categorical variables. Chi square test was used for comparison of frequency and proportion of the qualitative data. Student's t test and One-Way ANOVA were used for the comparison between two and more than two groups, respectively, regarding the quantitative data. In order to examine the association between variable correlations, the co-efficiency test was used. All statistical analysis was performed with SPSS Software and P value less than 0.05 were considered as significant.

## 4. Results

A total of 840 healthy subjects were evaluated for determination of anti-HBsAb. Among these, 43.5% (365/840) were males and 56.5% (475/840) were females. Their age ranged from 1 to 18 years with a mean age of 9.8 ± 5.2 years; 226 children (26.9%) were aged 1 to 5 years, 245 ones (29.2%) were aged 6 to 10 years, 192 subjects (22.9%) were aged 11 to 15 years, and 177 adolescents (21.1%) were aged 16 to 18 years.

The overall rate of protection among studied subjects was 55.2% (464/840); the proportion rate for males and females was 57% (208/475) and 53.9% (256/365), respectively. Table 1 shows the distribution of the children according to their protection status by gender and age groups. No significant difference was detected between males and females (P = 0.20). Significant difference was detected among age groups and protective anti-HBsAb level and geometric mean titer (GMT). The protection rate and mean level of anti-HBsAb were decreased significantly with increasing age (P = 0.0001). The prevalence of protective anti-HBsAb levels in the third age group (11-15 years) was at the lowest level (40.1% with GMT equal to 53.5 ± 131.5). As the age of children and adolescents was increasing, there was a significant decrease in both protection rate and GMT (P = 0.0001).

**Table 1.** Prevalence of Anti-HBsAb by Gender and Age Groups

	Anti-HBsAb < 10 IU/L, No. (%)	Anti-HBsAb ≥ 10IU/L, No. (%)	GMT, IU/L, mean ± SD
**Gender**			
Male	157 (43)	208 (57)	130.4 ± 266.6
Female	219 (46.1)	256 (53.9)	109.3 ± 217.2
**Age groups, y**			
1 to 5	63 (27.9)	163 (72.1)	202 ±336.5
6 to 10	107 (43.7)	138 (56.3)	118.6 ± 223.7
11 to 15	115 (59.9)	77 (40.1)	53.5 ± 131.5
16 to 18	91 (51.4)	86 (48.6)	82.2 ± 160.1
**Total**	376 (44.8)	464 (55.2)	118.5 ± 239.5

Protective antibody levels were detected in 90% of the children, one year after vaccination. They were decreased thereafter to 63.6%, 54.2%, 35.7%, and 48.9% after five, ten, fifteen, and eighteen years of vaccination, respectively. The mean level of anti-HBsAb was 272.35 IU/L one year after vaccination. It was declined to 85.9 IU/L, 95.1 IU/L, 35.7 IU/L and 91.4 IU/L after 5, 10, 15 and 18 years of vaccination, respectively.

**Figure 1.**
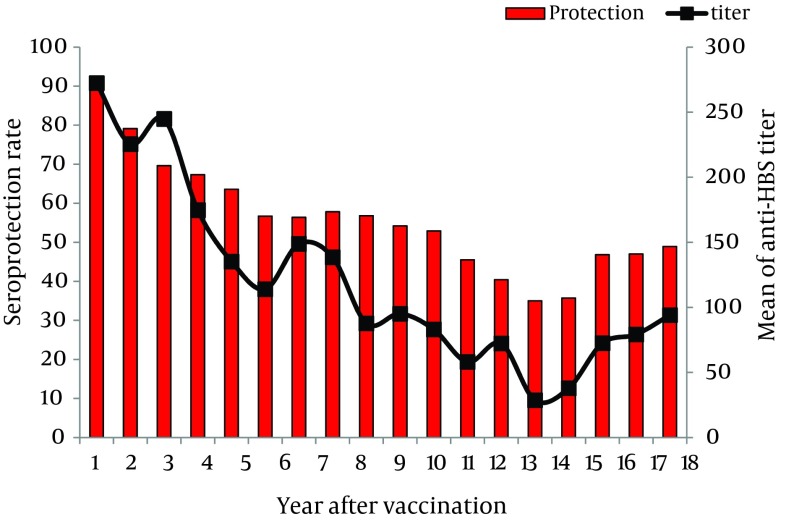
Distribution of Seroprotection and Anti-HBsAb Titer 1 to 18 Years After Vaccination Among the Study Population

The linear curve of declining trend of immunity to HBV and anti-HBsAb titer (GMT) over time was shown in Figure 1. Statistically significant negative correlations were found between immunity to HBV and age (r = -0.220, P = 0.0001) and also between anti-HBsAb titer (GMT) and age (r = -0.224, P = 0.0001). Seroprotection rates decreased significantly with increasing age due to worn down anti-HBsAb titers over the time (P < 0.001).

**Figure 2.**
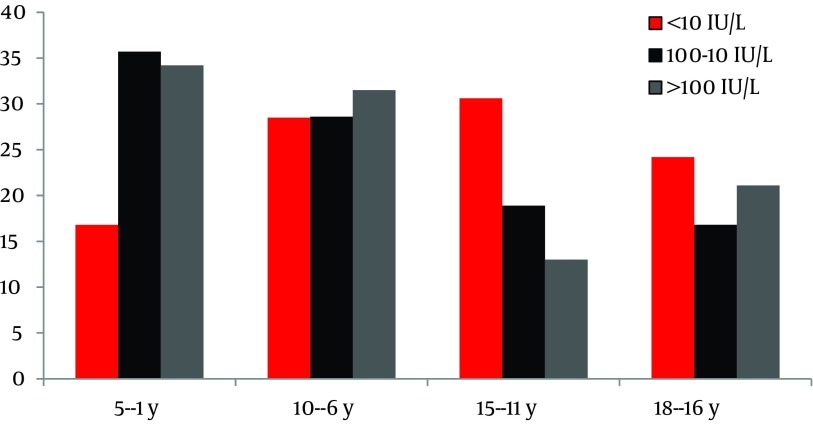
Distribution of Anti-HBsAb Levels in Different Age Groups

Regarding classification of antibody titers in three groups as less than 10 IU/L, between 10 IU/L and 100 IU/L and more than 100 IU/L, the rate of protective levels of anti-HBsAb among weak responders was 280/840 (33.3%) and in good responders was 184/840 (21.9%). Figure 2 shows the distribution of anti-HBs antibody levels in different age groups.

## 5. Discussion

This was the first study to evaluate in details the long term antibody persistence against HBV among 1 to 18 years old vaccinated population in Ahvaz, southwest of Iran. In this study we examined the persistence of anti-HBsAb levels in children and adolescents that had been immunized against HBV in their first year of life, 18 years after the implementation of a nationwide HBV vaccination program which had been commenced in 1993. A potential problem of HBV immunization is that vaccine-induced anti-HBsAb titers diminish to low or undetectable levels with age.

In our study, protective antibody level against HBV was detected in 90% of children one year after vaccination, which was then found to be declined significantly over time to 48.9% in 18 years old vaccinated population. Similarly, an Egyptian study for evaluation of efficacy of vaccination among 242 children reported seroprotection rate at one year and at 6 to 11 years after vaccination to be 84.3% and 39.3%, respectively (19).

Our findings revealed the potential problem of HBV immunization that vaccine-induced anti-HBsAb levels might decline to low levels with age. A previously performed study in Iran reported that 47.9% of children had protective levels of anti-HBsAb levels 10 years after vaccination (20). Also another study in Iran reported positive response rate of 75.4% among children in Ahvaz 5 years after HBV vaccination (21). Moreover, the results of another study in Isfahan showed protective anti-HBsAb levels in 29.2% of children 6 years after vaccination (22).

In a series of studies among healthy children, who had received a complete HBV immunization program, protective anti-HBsAb levels were gradually declined after the last dose of vaccine (23-26).

A similar study in Saudi Arabia showed positive response rate of 38% among students 18 years after vaccination (22). In another study on Egyptian natives, the seroprotective anti-HBsAb level was detected to be 47.5 % at 6 to 7 years, and 39% at 9 year after primary vaccination (27). Similarly the results of a study in Taiwan showed that the frequency of children with seroprotective levels of anti-HBsAb was gradually decreased from 71.1% at the age of 7 years to 37.4% at the age of 12 years (28). Serologic studies have shown that the level of anti-HBsAb would drop within the first few years after vaccination and that a third to half of vaccinated children may have titers below 10 IU/L by 10 to 15 years of age. They also have reported low response to booster among 67% of 5-year-old children, 52% of 9-year-old children, and 44% of 15-year-old children (29).

Some researchers reported that the rate of persistence of anti-HBsAb of vaccinated individuals had been decreased by age (17, 18). The declining trend of anti-HBsAb levels which was reported in this study and the diversity of results in different studies may be largely attributed to differences in the environmental and genetic factors, type and dose of the vaccines, age of initial vaccination, schedule of immunization, and intervals between vaccine administrations.

Regarding the duration of immunity in children, researchers have suggested that universal vaccination of infants in the first and eleventh years of their life may lead to improvement of the endemic status of infection in the general population (30).

Other findings of the present study demonstrated that linear decline of the protective anti-HBsAb titers among the vaccinated population occurred over the time from 90% to 48.3% after 18 years from primary vaccination. This decline was further conﬁrmed by the decline in the GMT level over the past 18 years in our region (Figure 2). These ﬁndings were consistent with the results of previous studies from Saudi Arabia, China, Taiwan, Hong Kong, and Alaska (22, 31-34).

An important finding of this study was that the protective anti-HBsAb levels at the age of 14 years were declined dramatically and reached its minimum which is a warning because this is the age of puberty associated with risky behaviors. Therefore, the Center for Disease Control and Prevention must determine anti-HBsAb levels in different areas and administrate a booster dose of HBV vaccine at this age.

The results also showed that the protective anti-HBsAb levels were higher in individuals at the age of 18 years compared to 15 year-old adolescents. This was due to the implementation of universal HBV vaccination of individuals aged 18 to 25 years in Iran from 2002 according to the vaccination schedule which was approved by the National Committee of Hepatitis (5).

Regarding to low levels of protective HBV antibody during adolescence, some investigators suggested the use of a booster dose of vaccine for adolescents to increase the immunity rate against HBV during adulthood (17-19). Determination of anti-HBsAb levels among children after vaccination is also recommended. Several studies have demonstrated that the booster dose of HBV vaccine provides long-term immunity against HBV infection (31, 32).

The results of our study in comparison with other studies revealed no significant difference regarding gender and anti-HBsAb levels (10, 34). A previous study found that anti-HBsAb production was not affected by sexual factors such as feminine hormones (10). Others have reported significant differences between anti-HBsAb levels in males and females (12, 22). This assumption could be supported by the results of our observation and other previous studies.

In conclusion, after 1 and 18 years of primary vaccination with recombinant HBV vaccine, 90% and 48.9% of the vaccinated population had protective levels of anti-HBsAb, respectively. The findings demonstrated a decline in anti-HBsAb titers over the time after vaccination. Further studies, especially cohort ones, are recommended to determine the duration of HBV vaccine protection and the necessity of booster doses.
